# Tumour-associated neutrophils orchestrate intratumoural IL-8-driven immune evasion through Jagged2 activation in ovarian cancer

**DOI:** 10.1038/s41416-020-1026-0

**Published:** 2020-08-11

**Authors:** Moran Yang, Guodong Zhang, Yiying Wang, Mengdi He, Qing Xu, Jiaqi Lu, Haiou Liu, Congjian Xu

**Affiliations:** 1 Shanghai Key Laboratory of Female Reproductive Endocrine Related Diseases, Obstetrics and Gynecology Hospital, Fudan University, 200011 Shanghai, China; 2 Department of Biochemistry and Molecular Biology, School of Basic Medical Sciences, Fudan University, 200032 Shanghai, China; 3 Department of Gynecology, Second People’s Hospital, Kashgar, Xinjiang Uyhur Autonomous Region, 844000 Xinjiang, China

**Keywords:** Cancer microenvironment, Preclinical research

## Abstract

**Background:**

Tumour associated neutrophils (TANs) play a controversial role in regulating immune surveillance and immune evasion in various malignancies. Here, we investigated the relevance of TANs with the prognosis and immune microenvironment of epithelial ovarian cancer (EOC).

**Methods:**

We characterised TANs using flow cytometric analysis and immunofluorescence analysis. The prognostic merit of TANs in EOC was evaluated using cox regression analysis. Furthermore, we explored the therapeutic merit of targeting Notch signalling in EOC and determined its involvement in the immune microenvironment.

**Results:**

High level of TANs is associated with a dismal prognosis and immune tolerance in EOC. TANs impaired cytotoxic effects of CD8^+^ T cells partly through Jagged2 (JAG2). Notch pathway blocked using γ-secretase inhibitor LY3039478 and anti-JAG2 antibody led to retarded tumour growth and augmented cytotoxic effects of CD8^+^ T cells. IL-8 contributes to the recruitment of TANs and the induction of JAG2 expression in TANs. Blockade of CXCR2 signalling reduces tumour growth rate, accompanied by a decreasing amount of TANs and increasing activity of CD8^+^ T cells. JAG2^+^TANs is an independent predictor of clinical outcomes.

**Conclusion:**

JAG2^+^TANs are closely linked to IL-8-driven immune evasion microenvironment and may serve as a promising therapeutic target for the reinvigoration of anti-tumour immunity.

## Background

Epithelial ovarian cancer (EOC) is the seventh most diagnosed cancer among women worldwide and the most deadly gynaecological cancer.^1^ In 2018, ~22,240 women and 14,070 deaths were diagnosed with EOC in the United States.^2^ Standard treatment for EOC includes surgical debulking followed by platinum-based chemotherapy, which initially effectively controls the disease and succumbs to recurrent, chemoresistant disease in the absence of effective second-line treatment. Cancer immunotherapy has achieved unprecedented success in a large number of patients with multiple cancers and is conducting EOC testing.^3^ Clinical trials of immunotherapy (such as Keytruda, Opdivo, and Aavelumab)^4^ have produced ~10–15% relief in patients with advanced and recurrent EOC, and sometimes transient remission.^5^ Taken together, the relatively low response rate of immune checkpoint inhibitors warrant further investigation of the molecular and cellular mechanisms of EOC immune evasion.

The role of neutrophils in cancer is controversial because seemingly contradictory capabilities play a role in promoting tumour or anti-tumour effects.^6^ The tumour associated neutrophils (TAN) with a functional bias in N1 and N2 phenotype, cytokine repertoire, and its effects on CD8^+^ T cells has been observed.^7,8^ In murine studies, different tumour microenvironment factors, such as transforming growth factor-β (TGF-β) and granulocyte colony-stimulating factor (G-CSF) polarised TANs to the N2 phenotype.^7,9^ Whereas, blocking TGF-β or interferon-β (IFN-β) polarised TANs to N1 phenotype.^10^

In human studies, subpopulations of TANs can be polarised to an anti-tumour phenotype in response to low levels of granulocyte-macrophage colony-stimulating factor (GM-CSF) and IFN-γ in early lung cancer.^11^ Moreover, TANs isolated from colorectal cancer have anti-tumour properties.^12^ In contrast, tumour microenvironment cues polarise TANs to an immunosuppressive phenotype in the late stage of cancer.^13,14^ Therefore, the immunomodulatory phenotype of TAN depends in part on tumour stage and type. In the case of EOC, neutrophils coordinate with TNF-α/IL-17 signalling to promote ovarian cancer progression,^15^ as well as facilitate ovarian cancer premetastatic niche formation through neutrophil extracellular traps (NETs).^16^ Moreover, neutrophils obtained an inhibitory phenotype in response to supernatants from the ascites of newly diagnosed advanced EOC patients.^17^ However, the role of TANs in regulating T-cell responses in EOC remains elusive.

Neutrophils regulate immune responses involve a variety of mechanisms, including the release of chemokines/cytokines, NETs, recruitment of regulatory T cells (Tregs), and PD-L1/PD-1 interactions.^8,13,18^ However, the detailed mechanism of the complex interaction between neutrophils and T cells in the tumour microenvironment remains largely unknown. The Notch signalling pathway regulates the anti-tumour activity of CD8^+^ T cells.^19^ Activation of Notch1 or Notch2 directly regulates the transcription of effector molecules such as IFN-γ and granzyme B.^20,21^ Consistent with this, activation of Notch2 signalling by DLL1 overexpressing antigen presenting cells or clustered DLL1 enhances anti-tumour cytotoxicity and inhibits tumour growth.^22–24^ On the other hand, overexpression of Jagged-1 provides an inhibitory signal in CD8^+^ T cells, whereas treatment with anti-Jagged1/2 blocking antibody reactivates myeloid-derived suppressor cells (MDSCs)-mediated T-cell suppression in tumours.^25,26^ The Notch signal regulates the immunosuppressive microenvironment by acting on stromal cells (such as MDSCs and macrophages) and directly modulating the cytotoxic activity of CD8^+^ T cells.^19^ Further studies are needed to determine the effect of Notch ligands expressed in the TANs on the cytotoxic capacity of CD8^+^ T cells.

Pre-treatment neutrophilia and elevated neutrophil to lymphocyte ratio were significantly associated with poor prognosis.^27,28^ However, the clinical significance of TANs infiltrating EOC is still unclear and underlying functional mechanisms remain to be elucidated. In the present study, we have analysed the prognostic significance of EOC-infiltrating CD66b^+^ neutrophils by using a tissue microarray (TMA) in two cohorts. In addition, we have comparatively evaluated the effects of neutrophils from cancerous ovarian tissues and peripheral blood from healthy donors (HD) exposed to conditional media from EOC cell lines or cancerous ovarian tissues on the CD8^+^ T-cell responses. We further evaluated the role of Notch signalling in the immunosuppressive activity of TANs. Finally, the prognostic relevance of JAG2^+^TANs infiltration in EOC was explored.

## Methods

### Patients

We retrospectively collected 322 paraffin-embedded biopsy tissues from patients with EOC who underwent surgery at Gynecology and Obstetrics Hospital of Fudan University (Shanghai, China) between January 2008, and December 2015. However, only 290 of the 322 patients had comprehensive information about clinicopathological data and survival outcomes for complete analysis. In this study, 13 dots on the TMA were lost after immunohistochemistry. Consequently, we included 277 patients in this study. Computer-generated random numbers were used to assign these samples into a training cohort consisting of 144 samples and an internal validation cohort consisting of 133 samples. None of the patients had an autoimmune disorder or a history of prior cancer. None of the patients was treated with anti-tumour therapy prior to tumour resection. The available clinical characteristics of these patients are summarised in Supplementary Table 1. All patients were followed up until Apr. 2019. Fresh EOC tissues and autologous peripheral blood were obtained from 38 patients with EOC who underwent surgical resection at our hospital (Supplementary Table 2). Peripheral blood from 20 healthy donors (HDs) was used as control.

### Immunohistochemistry

Immunohistochemical staining was performed as described previously.^29^ Sections were incubated overnight in a humidified chamber at 4°C with mouse anti-human CD66b (1:600, Biolegend) and IL-8 antibodies (1:400, Sigma-Aldrich), respectively. HRP-conjugated secondary antibodies (Vectastain universal quick Kit) were applied for 30 min and visualised with 3′3-diaminobenzidine (DAB) (Vector Laboratories). Staining with isotype antibody was used as a negative control. Images were taken on the Nikon Eclipse 80i microscope (Nikon). All the staining cores were scored by two investigators (Y.W. and M.H.) independently blinded to treatment group when scoring, as well as blinded to patient outcomes throughout the study. The neutrophil infiltration was determined as the number of cells/high power field (HPF) using an algorithm developed for the National Institutes of Health (NIH) software ImageJ. Quantification was undertaken for IL-8 according to the intensity of staining in the tumour ranked from 0 to 3 (0 = no staining, 1 = low staining, 2 = intermediate staining, and 3 = high staining) and the percentage of area staining was calculated using an algorithm developed for NIH ImageJ software. H-Scores were calculated for each core by multiplying intensity score by the percentage of core staining and a median H-score calculated for all cores from each patient. Co-localisation of CD66b and JAG2 was performed using an immPRESS^®^ Duet Double Staining HRP/AP polymer kit following the instructions from the manufacture (Vector Laboratories). CD66b was visualised using the DAB Substrate which develops brown colour. JAG2 was visualised using the Red Substrate which develops magenta colour (Supplementary Fig. 1). The cut-off for each marker was calculated using a cut-off finder with the incorporation of the Log-rank method.

### Definition of cut-off values

For CD66b+ TAN density, ≤10 per HPF was defined as low infiltration and >10 per HPF was defined as high infiltration. For JAG2^+^TAN density, ≤2 per HPF was defined as low infiltration and >2 per HPF was defined as high infiltration.

### Preparation of single cells from EOC tissues

Fresh tissues were washed three times with PBS containing 1% foetal bovine serum before being minced into small pieces. The specimens were collected in RPMI-1640 medium containing 1 mg/mL collagenase IV and 10 mg/mL deoxyribonuclease I and mechanically dissociated using MACS Dissociator (Miltenyi BioTech). Dissociated cell suspensions were further incubated for 1 h at 37°C under continuous rotation. The cell suspensions were then filtered through a 40μm cell strainer (BD Labware). TANs were purified by using anti-CD66b magnetic beads (Miltenyi BioTech). Peripheral blood neutrophils (PBNs) from healthy donors and autologous EOC patients were isolated by EasySep Human Neutrophil Enrichment Kit (Stemcell Technologies). The purity of isolated TANs and PBNs were evaluated by flow cytometry for neutrophil markers CD66b and exceeded 80 and 95%, respectively, in cells used in functional assays. Peripheral blood CD3^+^ T cells were isolated from peripheral blood mononuclear cells (PBMC) were purified by using anti-CD3 magnetic beads (Miltenyi Biotec).

### Flow cytometry

Cell suspensions from human EOC tissues or ascites, and peripheral blood of HDs or patients with EOC, and subcutaneous tumours of B6C3F1 mice were stained with a panel of fluorochrome-tagged antibodies (Supplementary Table 3). Red cells were lysed with an ammonium chloride solution and samples were incubated with Live/Dead Fixable Dead Cell Staining Kit (Invitrogen) prior to staining to permit the identification of live cells. Samples stained with isotype-matched antibodies were used as negative controls. For sample acquisition, a Beckman Coulter cytoflex flow cytometer with FACS CytExpert software (Beckman Coulter) was used, and FlowJo software (Tree Star) used for the analyses.

### Preparation of supernatant-conditioned neutrophils

Tumour tissue culture supernatants (TTCS) were prepared by plating EOC tissues in 1 mL RPMI-1640 medium for 24 h. The supernatant was then centrifuged and harvested. Neutrophils from HDs were harvested and cultured with 50% TTCS for 12 h, and then washed with RPMI-1640 medium for three times. Alternatively, neutrophils from HDs were treated for 12 h with conditioned medium from ovarian cancer cells (OVCAR8, SKOV3,). Neutrophils cultured with RPMI-1640 medium were used as controls.

### In vitro neutrophil-T-cell co-culture system

In a 3-day incubation, bead-purified peripheral CD3^+^ T cells (1 × 10^5^ cells/well in 96-well plates) were labelled with carboxyfluorescein succinimidyl ester (CFSE) and co-cultured with autologous neutrophils isolated from tumours or conditioned medium blood neutrophils. For co-stimulation experiments, 96-well flat bottom culture plates were coated overnight with anti-CD3 (clone UCHT1, 10 μg/ml). Neutrophils and CD3^+^T cells were cultured at a different ratio in 200 μL RPMI-1640 medium containing rIL-2 (20IU/mL), and anti-CD28 (1 μg/mL) antibodies, with or without 1 μM LY3039478 (Notch signalling inhibitor). When indicated, co-culture experiments were performed by using Transwell plates with a pore size of 0.4 μm (Corning, MA). After 3-day incubation, the cells were harvested for flow cytometry analysis.

### Immunofluorescence

Tissues were immediately frozen and embedded in OCT compound embedding medium for immunofluorescence staining. Slides were incubated with mouse anti-human CD66b (1:200) and rabbit anti-human CD8 (I:200; Invitrogen) at 4 °C overnight. Alexa Fluor 488- or 594-conjugated donkey anti-mouse/rabbit secondary antibodies were used (Jackson Immuno Research Labs). Slides were mounted using Vectashield w/DAPI (Vector Laboratories). Images were taken using a confocal microscope (TCS SP5; Leica Microsystems, Buffalo Grove, IL).

### Soluble Jagged2 ligand immobilisation and T-cell stimulation

Soluble Jagged2 ligand immobilisation was performed as described previously.^30^ The recombinant human Jagged2-Fc fusion chimera (R&D Systems) was dissolved in phosphate-buffered saline and immobilised in flat-bottom 96-well plates for 20 h at 4 °C, at 10 μg/mL (100 μL/well). As a control for Jagged2, human IgG-Fc (R&D Systems) was used. Plates coated with Jagged2 or IgG-Fc were washed and then coated overnight with anti-CD3 (clone UCHT1, 10 μg/ml). CD3^+^T cells were cultured in 100 μL RPMI-1640 medium containing rIL-2 (20IU/mL), and anti-CD28 (1 μg/mL) antibodies, with or without 1 μM LY3039478. After 3-day incubation, the cells were harvested for flow cytometry analysis.

### RNA isolation and quantitative reverse-transcription PCR

We extracted total RNA from FACS isolated CD3^+^T cells using TRIzol reagent (Invitrogen) following the manufacturer’s instructions. mRNA expression was quantified by quantitative reverse-transcription PCR using an SYBR PrimeScript reverse-transcription PCR kit (Takara Bio). The forward primer for HES-1: 3′-CATCTGAGCACAGAAAGTCATCAA-5′, the reverse primer for HES-1: 3′-CGAGCTATCTTTCTTCAGAGCAT-5′. The forward primer for Glyceraldehyde-3-phosphate dehydrogenase (GAPDH):3′-CAGCCTCAAGATCATCAGCAATG-5′, the reverse primer for GAPDH: 3′- CATGAGTCCTTCCACGATACCA-5′. GAPDH was used as an internal control.

### Mice and in vivo studies

Female B6C3F1 mice (4–6 weeks old; ∼25 g body weight) were obtained from Charles River Laboratories (Beijing, China). Mice were housed in standard ventilated cages with a maximum of five animals per cage and were placed at 24 ± 1°C in light-controlled SPF room (light:6:00–18:00, dark:18:00–6:00), and a relative humanity of 55% ± 5%. The mice were randomly divided into two groups. Mice were acclimatised for a week before implantation of tumours. Mice were anesthetised by inhalation of isoflurane mixed with oxygen using an anaesthesia machine (Reyward Life Technology, China). Isoflurane inhalation was chosen because of its faster anesthetised state and recovery, minimal effect on cardiovascular function and metabolism. A murine ovarian cancer cell line OV2944-HM-1 was obtained from the RIKEN BioResource Center (Japan). HM-1 tumour cells (1 × 10^6^ cells) were re-suspended in 100 μL ice cold PBS and injected subcutaneously in the right flank of B6C3F1 mice (five mice/ group) under anaesthesia using 27 G needle in a biosafety cabinet. Tumour growth was monitored daily and measured every 2–3 days using callipers. Tumour volume was determined as length (mm) × width^2^ (mm^2^) × 0.5. For Notch signalling inhibition, LY3039478 was administered via oral gavage formulated in 1% Na-CMC, 0.25% Tween 80, and 0.05% anti-foam. Dosing was done at 8 mg/kg three times per week. The vehicle was used as control. Jagged2 neutralisation was described as previously.^31^ Mice received intraperitoneal injections of anti-Jagged2 (BioXCell, 250 μg) or IgG as control at days 4, 6, 8, 10, 12, 14 after subcutaneous injection of HM-1. For CXCR2 inhibition, mice received twice a week intraperitoneal injection of SB225002 (Selleck, 1 mg kg^−1^). Mice were killed by carbon dioxide inhalation followed by the tumours were harvested. All mouse experiments complied with the Guidelines for Animal Health and Use (Ministry of Science and Technology, China, 2006). Animal experiments were approved by the Institutional review board and the ethics committee of Fudan University.

### Ex vivo tumour inhibition assay

Ex vivo tumour inhibition assay was performed as described previously.^32^ Briefly, single cell suspension was cultured in 3D assay medium (RPMI-1640 medium prepared with 2% Matrigel (BD) and supplemented with 100 IU/mL rhIL-2, 100 U/mL penicillin, 100 μg/mL streptomycin and 10% foetal bovine serum) in the presence or absence of 1 μM LY3039478 for 72 h at 37 °C and 5% CO_2_. Cells were collected by digestion with Liberase DH (Sigma Aldrich) and subjected to phenotypic analysis by flow cytometry as above.

### Enzyme immunoassay for IL-8 detection

Human EOC tissues (wet weight, 10–20 mg) were homogenised in ice-cold 0.1 M phosphate buffer with protease inhibitor cocktails (Sigma Aldrich, St. Louis, MO) and centrifuged at 12,000×*g* for 10 min at 4 °C. The protein concentration of samples was measured by BCA protein Assay kit (Thermo, RD) to standardise IL-8 levels. Human EOC ascites was also collected. IL-8 levels in the samples were determined by an IL-8 ELISA kit (Biolegend).

### Neutrophils chemotaxis assay

We tested neutrophils chemotaxis using a Transwell system (Corning) with 5 mm polycarbonate membranes. PBNs suspended in RPMI-1640 containing 2% foetal bovine serum (1 × 10^5^ cells/100 μL) were added to the upper wells and incubated for 24 h at 37 °C, and 5% CO_2_. Supernatants from EOC with or without control Ab (1 μg/mL), anti-CXCL1/2/3 (1 μg/mL), anti-CXCL5 (1 μg/mL), anti-IL-8(1 μg/mL), and SB225002 (CXCR2 inhibitor) were added to the lower chamber. Cells in the lower chamber were collected and analysed for CD66b using flow cytometry (Biolegend).

### TCGA analysis

TCGA-OV ovarian cancer mRNA expression data from 395 cases with clinical data were downloaded from the GDC legacy archive (https://protal.gdc.cancer.gov/) in Nov 2017. The immune cell proportions from TCGA were analysed by CIBERSORT as described previously^33^ (http://cibersort.stanford.edu/) with the algorithm using the LM22 signature and 1000 permutations. The optimal cut-off values for a proportion of neutrophils were defined as the point with the most significant (log-rank test) split and calculated using the web-based tool “cut-off finder” (http://molpath.charite.de/cutoff/) for the entire cohort.

### Statistics

Graphs were made and statistical analyses were performed using Prism software (GraphPad Software Inc.). The significance of differences between two groups was calculated with a two-sided Mann–Whitney *U*-test. The significance of differences between groups was determined by a non-parametric Kruskal–Wallis test, followed by Dunn’s post-hoc analysis. For ex vivo tumour inhibition test, paired Student’s t-test was used for comparison of mean between two groups. The correlation between two groups was assessed using Spearman’s rank analysis. For human subjects, statistics comparing different parameters were determined by Chi-square test, whereas progression free survival and overall survival were determined by Kaplan–Meier test. Multivariable analyses were using the Cox proportional hazards model to correct clinical covariates including age, residual tumours, and tumour stage for all survival analyses in our study. The data were represented as mean ± SDs. *P* values were considered significant if *P* < 0.05.

## Results

### Tumour associated neutrophils correlate with poor clinical outcomes in EOC

**Fig. 1:**
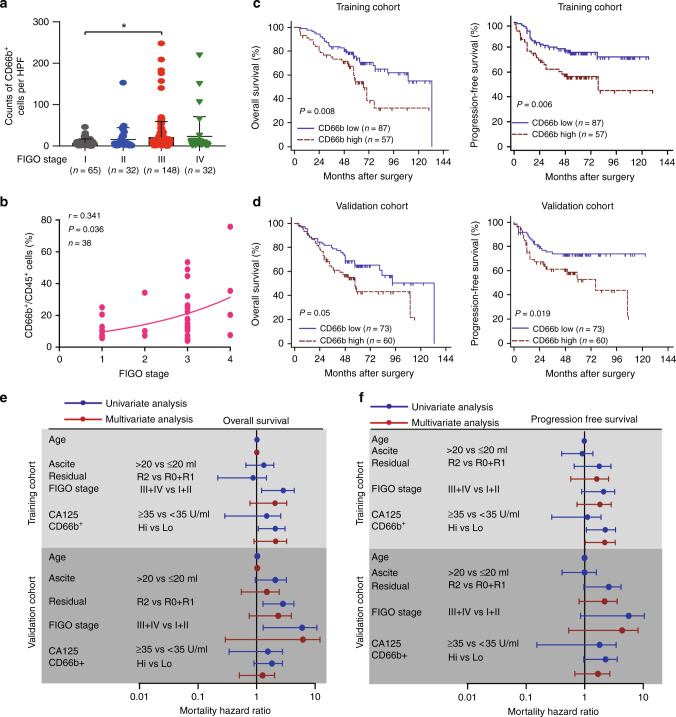
Tumour associated neutrophils associate with poor clinical outcomes in EOC. **a** The counts of CD66b per high power field (HPF) were compared between different FIGO stages in EOC patients (*P < 0.05). **b** Patient disease stage compared to the proportions of CD66b^+^ cells to CD45^+^ cells from EOC tissues (*n* = 38). A*n* exponential growth equation is shown. **c** Kaplan–Meier plots for overall survival and progression free survival of EOC patients based on CD66b expression detected by immunohistochemistry (IHC) in the training cohort. **d** Kaplan–Meier plots for overall survival and progression free survival of EOC patients based on CD66b expression in the validation cohort. **e**, **f** Cox univariate and multivariate analyses identified the independent prognostic factors for overall survival (**e**) and progression free survival (**f**) in EOC patients from the training cohort and validation cohort.

To elucidate the influence of TANs on clinical outcomes, we performed immunohistochemical analysis of CD66b in TMAs and quantified (Supplementary Fig. 1). The density of TANs has been associated with tumour stage (Fig. 1a). Consistently, the proportions of CD66b^+^ cells increased along with increased disease stage (spearman *r* = 0.341) (Fig. 1b). We performed survival analysis for high and low levels of CD66b expression and observed a significant association of the high CD66b densities with significantly decreased overall survival (OS) and progression free survival (PFS) (*P* = 0.008 and *P* = 0.006) in the training cohort, resulting in a hazard ratio of 2.178 and 2.349 (Fig. 1c and Supplementary Table 4). Furthermore, we found a correlation between high CD66b densities and significantly unfavourable PFS, but not significantly OS in the validation cohort (Fig. 1d). Cox multivariate analyses indicated that TANs may not serve as an independent prognostic factor to predict unfavourable survival of patients with EOC (Fig. 1e, f and Supplementary Table 4). Consistently, we also found a significant relationship between OS and neutrophils abundance inferred via CIBERSORT from TCGA; median OS 54.04 months (95% CI 47.51–58.05) compared with high neutrophils median OS 41.53 months (95% CI 38.01–48.75) (*P* = 0.002) (Supplementary Fig. 2a).In a multivariate analysis that included age, platinum status, residual tumours, and FIGO stage as confounders, the hazard ratio (HR) for disease mortality among patients with high neutrophils infiltration was 1.86 (95% CI 1.35–2.56) (*P* < 0.001) (Supplementary Fig. 2b and Supplementary Table 5). Also, EOC, as a heterogeneous tumour, is composed of different histological subtypes. Due to the limited number of endometrioid and mucinous ovarian cancer, we further investigated the clinical significance of TAN in serous and clear cell ovarian cancer patients. The high TANs densities indicated poor clinical outcomes in patients with serous ovarian cancer, but not in patients with clear cell ovarian cancer (Supplementary Fig. 3).

### Tumour associated neutrophils contribute to cancer immunosuppression through dampening CD8^+^ T-cell activity partly under a contact-dependent manner

**Fig. 2:**
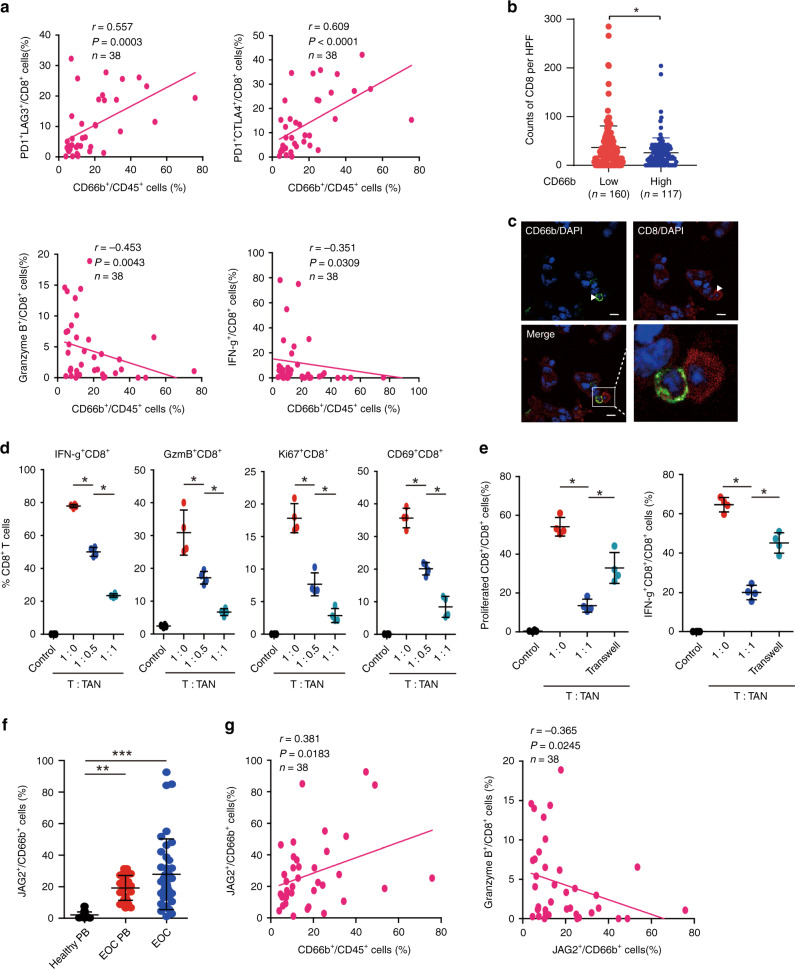
Tumour associated neutrophils suppress CD8^+^ T-cell responses partly under a contact-dependent manner. **a** The correlations assessed by Spearman correlation analyses between the proportion of TANs and inhibitory receptor-positive or effector molecule-positive CD8^+^ T-cells in specimens of EOC. **b** The counts of CD8 per HPF in each tissue of EOC patients were compared between CD66b high and low group (*P < 0.05). **c** CD66b/CD8 staining of EOC specimens. The right panel is merged by the aforementioned two images. (Bar = 50μm.) **d** Peripheral CD3^+^ T cells from EOC patients were co-cultured for 3 days with autologous TANs from EOC tissues. Statistical analyses of the proportion of effector molecule-positive cells to total CD8^+^ cells were shown (*n* = 4)(*P < 0.05). **e** Carboxyfluorescein succinimidyl ester (CFSE)-labelled peripheral CD3^+^ T cells of EOC patients were co-cultured for 3 days with or without autologous neutrophils from tumour tissues and in conditions preventing cell contact (Transwell). Statistical analyses of proportions of proliferated CD8^+^ T cells and IFN-γ^+^ cells to total CD8^+^ T cells were shown (*n* = 4) (*P < 0.05). **f** Statistical analyses of proportions of JAG2^+^ cells to CD66b^+^ cells in healthy peripheral blood (*n* = 20), peripheral blood of EOC patients (*n* = 26) and EOC specimens (*n* = 38) were shown (**P < 0.01, ***P < 0.001). **g** The correlations assessed by Spearman correlation analyses between the proportions of JAG2^+^ cells to CD66b^+^ cells and CD66b^+^ cells to CD45^+^ cells (left panel) or Granzyme B^+^ cells to CD8^+^ cells (right panel).

To investigate the influence of TANs on the cytotoxic capabilities of CD8^+^ T cells, we analysed the infiltration of CD8^+^ T cells in EOC. We observed a positive correlation between the proportion of CD66b^+^ cells and PD1^+^LAG3^+^CD8^+^, PD1^+^CTLA4^+^CD8^+^ T cells, whereas CD66b^+^ cells were negatively correlated with Granzyme B^+^CD8^+^ and IFN-γ^+^CD8^+^ T cells (Fig. 2a). In addition, through IHC analysis, we found that EOC with high TANs infiltration exhibited fewer CD8^+^ T cells (Fig. 2b). TANs co-localise with CD8^+^ T cells in tumour tissues, suggesting that TANs may inhibit CD8^+^ T-cell activity under a contact-dependent manner (Fig. 2c). In order to further validate the inhibitory role of TANs, we investigated the functions of TANs in vitro. TANs from specimens of EOC were isolated and co-cultured with purified autologous peripheral blood CD3^+^ T cells. T/TANs co-cultures showed that TANs inhibited activation of CD8^+^ T cells (CD8^+^IFN-γ^+^, CD8^+^GzmB^+^, CD8^+^CD69^+^) and CD8^+^ T-cell proliferation (CD8^+^Ki67^+^), indicating TANs display an immunosuppressive function (Fig. 2d). Consistent with above, the inhibitory effect of TANs on proliferation and activation of CD8^+^ T cells was partly relieved by isolating TANs from CD8^+^ T cells with transwell (Fig. 2e). Since the phenotype of CD8^+^ T cells is modulated by Notch signalling, the mechanisms by which neutrophils modulate activities of CD8^+^ T cells remain unknown. Interestingly, compared with PBNs from healthy donors, PBNs from EOC patients expressed significantly higher levels of JAG2, while TANs exhibited the highest level (Fig. 2f). Moreover, we found a significantly positive correlation between proportions of JAG2^+^ neutrophils and neutrophils per leukocytes, and a negative correlation between the proportions of JAG2^+^CD66b^+^ cells and GzmB^+^CD8^+^ T cells (Fig. 2g), indicating that JAG2^+^TANs may contribute to suppressing the activities of CD8^+^ T cells.

### JAG2^+^TANs suppress the cytotoxic activity of CD8^+^ T cells partly depends upon Notch signalling

**Fig. 3:**
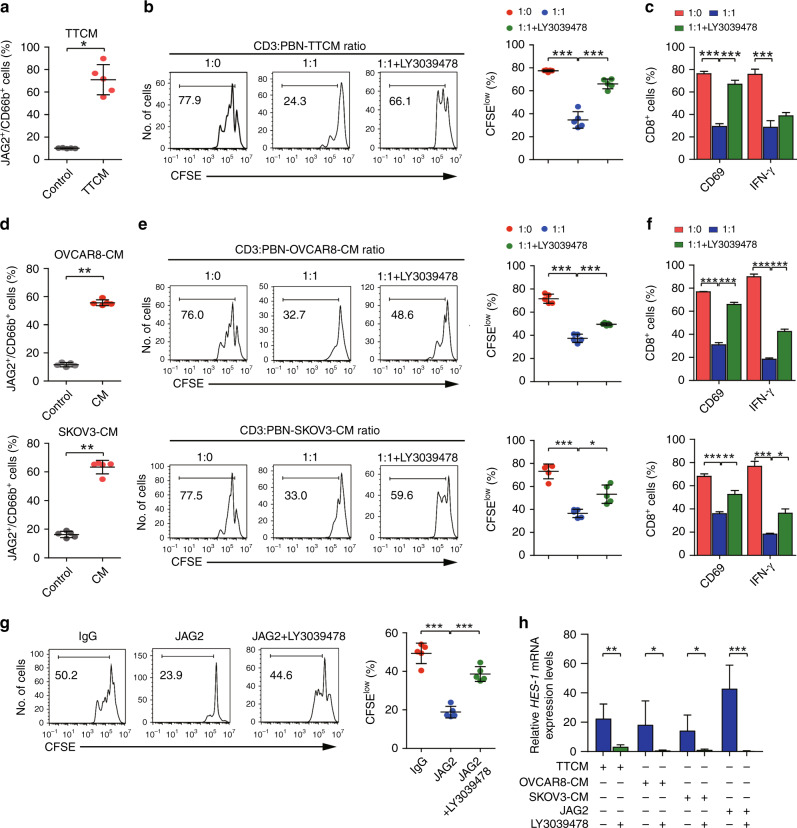
JAG2^+^TANs suppress the cytotoxic activity of CD8^+^ T cells partly depends upon Notch signalling. **a** Peripheral PBNs from healthy donors were cultured for 12 h with or without a 50% tumour tissue conditional medium (TTCM). Statistical analyses of proportions of JAG2^+^CD66b^+^ cells were shown (*n* = 5) (*P < 0.05). **b** Representative histograms of CD8^+^ T-cell proliferation at corresponding CD3^+^ to TTCM conditioned PBN cell ratio in the absence or presence of LY3039478 were shown, and quantification of CD8^+^ T-cell proliferation using CFSE dilution (right panel) (*n* = 5) (***P < 0.001). **c** Statistical analyses of proportions of effector molecule-positive cells to total CD8^+^ T cells were shown (*n* = 5) (***, *P* < 0.001). **d** Peripheral *P*BNs from healthy donors were cultured for 12 h with or without 50% ovarian cancer cell lines OVCAR8 (upper panel) and SKOV3 (lower panel) conditional medium. Statistical analyses of proportions of JAG2^+^CD66b^+^ cells were shown (*n* = 5) ^(^**P < 0.01). **e** Representative histograms of CD8^+^ T-cell proliferation at corresponding CD3^+^ to OVCAR8/or SKOV3 conditioned medium cultured PBN cell ratio in the absence or presence of LY3039478 were shown (left panel), and quantification of CD8^+^ T-cell proliferation using CFSE dilution (right panel) (*n* = 5) (*P < 0.05; ***P < 0.001). **f** Statistical analyses of proportions of effector molecule-positive cells to total CD8^+^ T cells were shown (*n* = 5) (*P < 0.05; ***P < 0.001). **g** Representative histograms of CD8^+^ T-cell proliferation at corresponding CD3^+^ cells exposed to immobilised soluble Jagged2 ligand (10 μg/mL) or IgG as a control in the presence or absence of LY3039478 were shown (left panel), and quantification of CD8^+^ T-cell proliferation using CFSE dilution (right panel) (*n* = 5) (***P < 0.001). **h** Statistical analyses of relative *HES-1* mRNA expression in corresponding cells were shown (*n* = 5) (***P < 0.001).

EOC tumour tissue conditional cultural medium (TTCM) significantly upregulated JAG2 expression in neutrophils compared with the control medium (Fig. 3a). Purified peripheral CD3^+^ T cells were co-cultured with TTCM conditioned allogeneic blood neutrophils. Consistently, TTCM conditioned neutrophils significantly suppressed proliferation and effective activities of CD8^+^ T cells, which were reversed by Notch inhibitor, LY3039478 (Fig. 3b, c). This suggests that TANs exhibit an inhibitory effect on CD8^+^ T cells in partly dependence of Notch signalling. Furthermore, we found that EOC cell lines (OVCAR8 and SKOV3) condition medium induced JAG2 expression in peripheral neutrophils (Fig. 3d). These neutrophils also suppressed proliferation and effective activities of CD8^+^ T cells, which were reversed by LY3039478 (Fig. 3e, f). In order to confirm the role of JAG2 in the JAG2^+^neutrophils induced immunosuppressive activity of CD8^+^ T cells. We found out JAG2 ligand directly suppressed the proliferation of CD8^+^ T cells (Fig. 3g). Consistently, JAG2 ligand and TTCM/or OVCAR8/SKOV3 CM-conditioned neutrophils induced upregulation of *HES-1* mRNA expression in lymphocytes (Fig. 3h), indicating that JAG2^+^TANs exert immunosuppressive activity partly dependent on Notch signalling.

### Notch inhibitor reinvigorates CD8^+^ T cells from tumour microenvironment infiltrated with high levels of JAG2^+^TANs

**Fig. 4:**
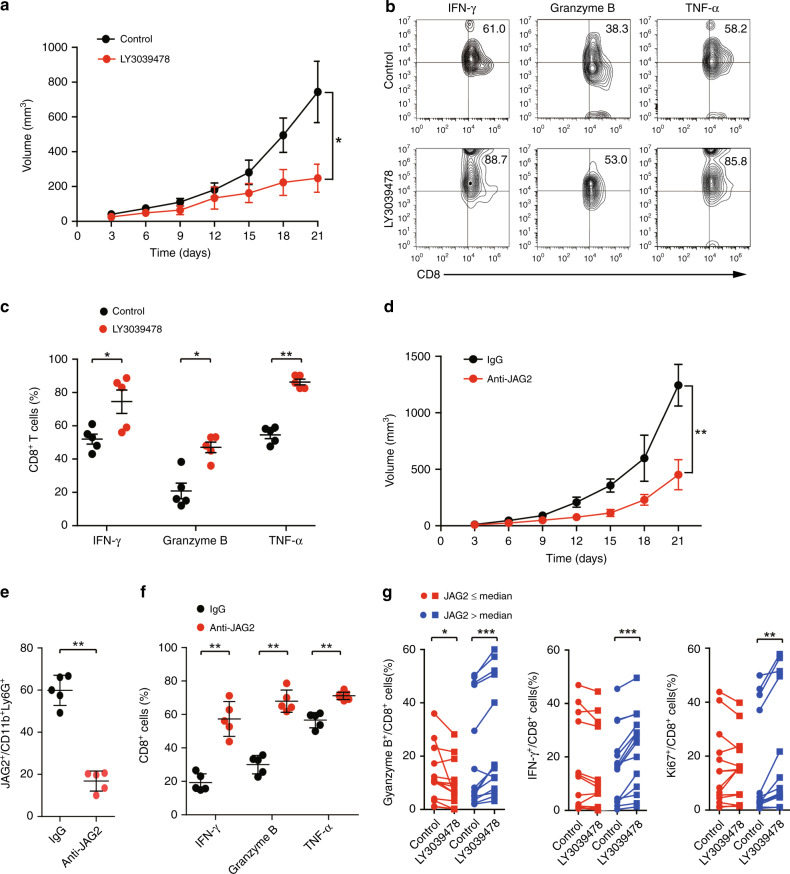
Inhibition of Notch signalling reinvigorates CD8^+^ T cells from tumour microenvironment infiltrated with high levels of JAG2^+^TANs. **a** Tumour growth curve in B6C3F1 mice subcutaneously injected with HM-1 cells and treated with LY3039478 or vehicle were shown (*n* = 5 per group) (*P < 0.05). **b** Representative flow cytometric plots of tumour infiltrating CD8^+^ T cells based on CD8 versus IFN-γ, Granzyme B, and TNF-α expression from control (upper) and LY3039478 treated group (lower) were shown (*n* = 5). **c** Statisti**c**al analyses of proportions of effector molecules-positive cells to total CD8^+^ T cells in corresponding groups of tumours were shown (*P < 0.05; **P < 0.01). **d** Tumour growth curve in B6C3F1 mice subcutaneously injected with HM-1 cells and treated with anti-JAG2 antibody or IgG were shown (*n* = 5 per group) (**P < 0.01). **e** Statistical analyses of proportions of JAG2^+^ cells to CD11b^+^Ly6G^+^ cells in the corresponding group of tumour tissues were shown (*n* = 5 per group) (**P < 0.01). **f** Statistical analyses of proportions of effector molecules-positive cells to total CD8^+^ T cells in corresponding groups of tumours were shown (*P < 0.05; **P < 0.01). **g** Statistical analyses of proportions of effector molecules-positive cells to total CD8^+^ T cells between EOC specimens infiltrated with low and high JAG2^+^TANs (distinguished by median proportions of JAG2^+^TANs) in the presence or absence of LY30394789 were shown (*n* = 14 per group) (*P < 0.05, **P < 0.01, ***P < 0.001).

To investigate the potential therapeutic value of LY3039478 in the treatment of EOC, we investigated whether LY3039478 inhibits tumour growth in vivo. We found that LY3039478 significantly retarded tumour growth in the subcutaneous transplantation EOC model (Fig. 4a). LY3039478 significantly enhanced IFN-γ^+^CD8^+^, Granzyme B^+^CD8^+^, and TNF-α^+^CD8^+^ T-cell proportions from tumour tissues compared with a control group (Fig. 4b, c). Consistently, the anti-JAG2 antibody also significantly inhibited tumour growth in vivo (Fig. 4d). The proportion of JAG2^+^TANs was significantly lower in the group treated with anti-JAG2 antibody, while the cytotoxic activities of CD8^+^ T cells were promoted (Fig. 4e, f). Furthermore, we applied LY3039478 to treat EOC tissues ex vivo. We found that LY3039478 potently enhanced cytotoxic activity of CD8^+^ T cells and increased the proliferation of CD8^+^ T cells in high level JAG2^+^TANs (JAG2^H^) infiltrating EOC tissues, but not in low level JAG2^+^TAN (JAG2^L^) infiltrating EOC tissues (Fig. 4g). These findings suggest that JAG2^+^TAN may serve as a potential biomarker for the treatment of EOC patients with LY3039478.

### IL-8 contributes to the recruitment of TANs and induction of JAG2 expression

**Fig. 5:**
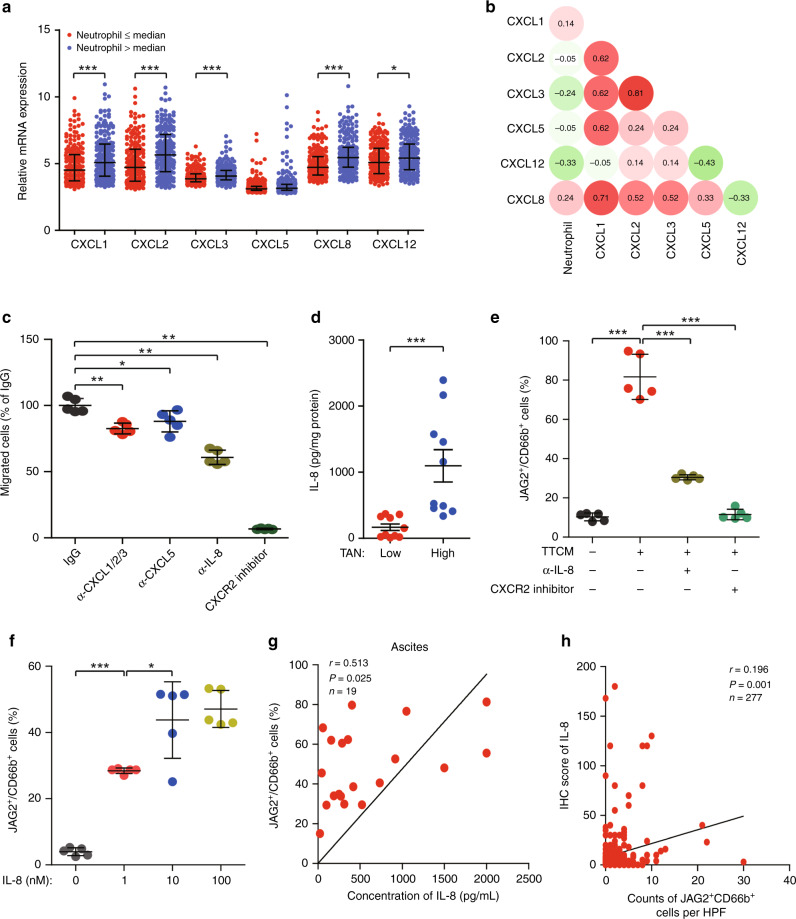
IL-8 contributes to the recruitment of TANs and induction of JAG2 expression. **a** Comparisons of mRNA expression of neutrophil chemoattractants (CXCL1, CXCL2, CXCL3, CXCL5, CXCL8, CXCL12) between EOC with low and high proportions of neutrophils were shown (calculated by CIBERSORT in Cancer Genome Atlas Cohort (*TCGA*) distinguished by median values) (*P < 0.05, ***P < 0.001). **b** Heatmap was shown for spearman’s correlation coefficients between neutrophil chemoattractants mRNA expression levels and proportions of neutrophils. **c** Statistical analyses of proportions of PBNs towards TTCM in the bottom chamber of transwell in the presence of α-CXCL1/2/3, α-CXCL5, and α-IL-8 neutralising antibody, as well as CXCR2 inhibitor were shown(*P < 0.05, **P < 0.01, ***P < 0.001). **d** Statistical analyses of IL-8 levels in EOC tissues with low or high neutrophils infiltration distinguished by median proportions of TANs were shown (*n* = 10 per group) (***P < 0.001). **e** Statistical analyses of JAG2^+^ cells to total CD66b^+^ PBNs exposed to TTCM for 12 h in the presence of α-IL-8 neutralising antibody or CXCR2 inhibitor were shown (*n* = 5) (***P < 0.001). **f** Statistical analyses of JAG2^+^ cells to total CD66b^+^ PBNs exposed to different concentrations of IL-8 for 12 h were shown(*n* = 5) (*P < 0.05; ***P < 0.001). **g** Correlation between proportions of JAG2^+^ cells to total CD66b^+^ cells and concentrations of IL-8 in ascites from EOC patients were shown (*n* = 19). **h** Correlations between counts of JAG2^+^TANs per high power field (HPF) and IHC score of IL-8 in EOC tissues microarray were shown (*n* = 277).

In order to determine the mechanism of neutrophils recruitment into the tumour, we screened neutrophil attracting chemokines (CXCL1, CXCL2, CXCL3, CXCL5, CXCL8 and CXCL12). We found that, except CXCL5, CXC chemokines in EOC with high neutrophils infiltration were significantly higher than that in EOC with low neutrophils infiltration from TCGA (Fig. 5a). Furthermore, we found that only CXCL8 mRNA expression was associated with a high proportion of neutrophils (Fig. 5b). Consistently, IL-8 neutralisation antibody and CXCR2 inhibitor significantly inhibited neutrophils migration towards tumour conditional medium (Fig. 5c). Higher concentrations of IL-8 were detected in high TANs infiltrating EOC tissues compared with low TANs infiltrating counterparts (Fig. 5d). In order to elucidate the effect of IL-8 on JAG2 expression in TANs, we applied anti-IL-8 neutralisation antibody and CXCR2 inhibitor to TTCM induced JAG2 expression in neutrophils. We observed both anti-IL-8 neutralisation antibody and CXCR2 inhibitor suppressed the TTCM-induced JAG2 expression in neutrophils (Fig. 5e). Consistently, we found that IL-8 induced JAG2 expression in neutrophils partly under a concentration-dependent manner (Fig. 5f). Interestingly, we also found a positive correlation between the proportion of JAG2^+^TANs and the concentration of IL-8 in the ascites of EOC patients (Fig. 5g). IHC analysis further confirmed the correlation between JAG2^+^TANs infiltration and IL-8 expression (Fig. 5h). This data suggests that IL-8 may be involved in attracting neutrophils into tumour microenvironment and induction of JAG2 expression.

### Blocking of IL-8 signalling with CXCR2 inhibitor enhances CD8^+^ T-cell activity

**Fig. 6:**
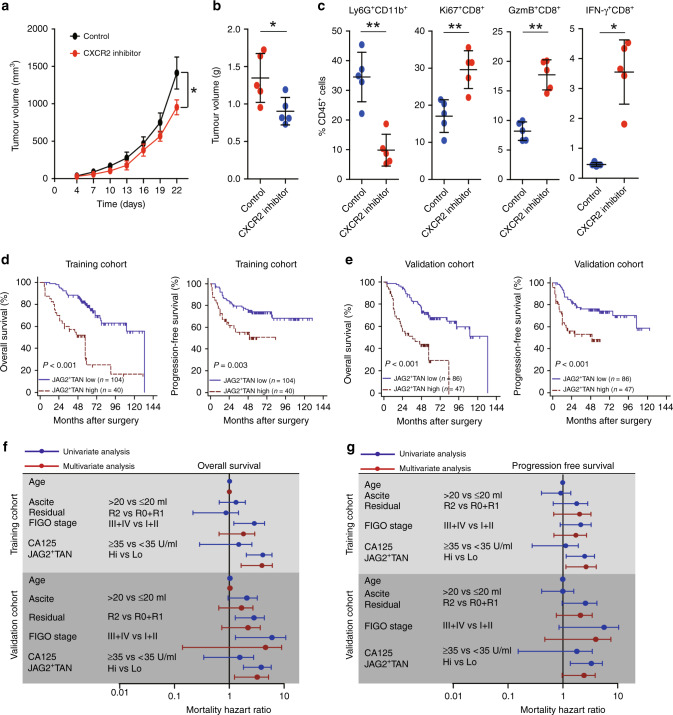
Blocking of IL-8 signalling with CXCR2 inhibitor enhances CD8^+^ T-cell activity. **a** Tumour growth curve in B6C3F1 mice subcutaneously injected with HM-1 cells and treated with CXCR2 inhibitor or vehicle were shown (*n* = 5) (*P < 0.05). **b** Tumour weights from B6C3F1 mice subcutaneously injected with HM-1 cells and treated with CXCR2 inhibitor or vehicle were shown (*n* = 5). **c** Statisti**c**al analysis of proportions of Ly6G^+^CD11b^+^ neutrophils, Ki67^+^CD8^+^ T cells, IFN-γ^+^CD8^+^ T cells, and GzmB^+^CD8^+^ T cells to total CD45^+^ cells in tumours from B6C3F1 mice treated with or without CXCR2 inhibitor were shown (*P < 0.05, **P < 0.01). **d** Kaplan–Meier plots for overall survival and progression free survival of patients with EOC based on JAG2^+^TANs counts in training cohort were shown. **e** Kaplan–Meier plots for overall survival and progression free survival of patients with EOC based on JAG2^+^TANs counts in the validation cohort were shown. **f**, **g** Cox univariate and multivariate analyses identified the independent prognostic factors for overall survival (**f**) and progression free survival (**g**) in EOC patients from the training and validation cohort.

To further ensure that IL-8 contributes to the recruitment of neutrophils into the tumour microenvironment, we applied the CXCR2 small molecule inhibitor SB225002 to subcutaneously transplanted EOC model. Consistently, the CXCR2 inhibitor retarded the tumour growth (Fig. 6a, b). CXCR2 inhibitor suppressed TANs recruitment, while increased Ki67^+^CD8^+^, GzmB^+^CD8^+^, and IFN-γ^+^CD8^+^ T-cells proportion (Fig. 6c). These findings suggest that blocking the CXCR2 pathway may enhance CD8^+^ T-cell response by retarding the recruitment of neutrophils.

### JAG2^+^TANs serve as an independent predictor of EOC patients

High infiltration of JAG2^+^TANs was found to be correlated with the FIGO stage of EOC patients (Supplementary Table 6). Furthermore, significant associations between high JAG2^+^TANs infiltration and poor OS and PFS were found in both training and validation cohort (Fig. 6d and e). Cox multivariate analyses indicated that JAG2^+^TANs may serve as an independent prognostic factor to predict unfavourable survival of patients with EOC (Fig. 6f, g and Supplementary Table 7). The high JAG2^+^TANs densities indicated poor clinical outcomes in patients with both serous and clear cell ovarian cancer (Supplementary Fig. 4).

## Discussion

Although tumour associated neutrophils are less prominent, the presence and importance of these cells have now attracted more attention.^8^ Here focusing on the role of TANs in the EOC immune evasion, we demonstrate that IL-8 can orchestrate immunosuppressive crosstalk between JAG2^+^TANs and CD8^+^ T cells. Treatment with a specific Notch inhibitor can enhance CD8^+^ T-cell activity in EOC infiltrating with high JAG2^+^TANs. This conclusion is supported by our observations that TANs, JAG2, IL-8 are cooperatively associated with poor clinical outcomes of EOC (Supplementary Fig. 5).

TANs promote immunosuppressive microenvironment by delivering soluble mediators such as arginase-1 (ARG1), interleukin-10 (IL-10), and reactive oxygen species (ROS), while little is known about the required cell contact signals.^34,35^ Notch has been implicated as a contact signal that regulates T-cell differentiation and activation.^34^ Notch ligands of the delta-like ligand family (DLL1, 3 and 4) promote Th1 and Th17 cell differentiation, while the jagged family (JAG1 and 2) induce Th2, Th9, and Treg differentiation.^36–38^ MDSC upregulates JAG1 and JAG2 expression via NF-κB-p65 signalling results in immunosuppression of CD8 T cells. Blocking Notch signalling with anti-JAG1/2 antibodies enhances CD8 T-cell responses and excites therapeutic effects in several cancer models.^26^ Our results are in line with this. TAN inhibits CD8^+^ T-cell activity, which can be reversed in vitro by the Notch inhibitor LY3039478. In addition, LY3039478 reactivates CD8^+^ T cells in EOC infiltrating with high JAG2^+^TANs ex vivo. How to modulate the cytotoxicity of CD8^+^ T cells by JAG2 expressed on TANs requires further investigation. JAG2 can modulate the immunosuppressive microenvironment by potentially competing with DLL ligands for CD8^+^ T cells, or potentially increase Treg cell generation.^38^ Furthermore, how tumour-derived signals reprogram neutrophils into subsets of JAG2^+^TAN deserves further investigation.

Though human neutrophils were found to produce cytokines that are crucial for B cells survival, maturation, and differentiation, defined as B‐cell‐helper neutrophils (NBH) and localised in the marginal zone (MZ).^39^ We found a slight depletion of memory B cells and Tfh cells in high proportion neutrophils group, indicating weakened humoral immunity.^40^ Since neutrophils engaged in complex regulation in the tumour environment,^6,8^ interactions occurring between neutrophils and adaptive immune cells such as B cells and Tfh cells can be mediated by distinct neutrophil populations.^13,41^

The oncogenic effects of Notch signalling in many malignancies lead to the development of targeted therapies, with different steps in their targeted pathways: γ-secretase inhibitors, antibodies targeting ligands or receptors, and compounds that target transcriptional activation. LY3039478 is an orally bioavailable γ-secretase inhibitor that is effective in a range of cancers in Phase 1 studies.^42^ EOC with high JAG2^+^TANs infiltration can be used as a marker for selecting patients for Notch targeted therapy. Since Notch signalling controls CD8^+^ T-cell priming and effector T-cell differentiation, cautions should be exercised in suppressing Notch signaling.^43^ Therefore, a better method should specifically target JAG1/2 by, for example, neutralising antibodies to reverse the immunosuppressive population and maintain functional effector T cells. Inhibition of Notch signalling by anti-JAG1/2 antibodies reduces the accumulation and tolerability of MDSCs in tumours, resulting in enhanced anti-tumour responses.^44^

IL-8 was originally discovered to be a potent attractor and activator of the polymorphonuclear leukocyte infiltrate, acting on CXCR1/2 and exerting its tumour-promoting function during cancer development.^45^ In studies of implanted EOC models, IL-8 is involved in cancer cell proliferation and angiogenesis.^46,47^ Previous study found that IL-8 is associated with decreased CD8^+^ T-cell infiltration and inhibits CD8^+^ T-cell function by inducing PD-L1 in gastric cancer macrophages.^48^ However, by promoting the recruitment of neutrophils and other monocytes to the tumour microenvironment, IL-8 is shown to be protective in EOC.^49^ In this study, we demonstrate that IL-8 contributes to the recruitment of neutrophils into the tumour microenvironment. Inhibition of CXCR1/2 signalling delays tumorigenesis and enhances CD8^+^ T-cell infiltration, consistent with previous results.^50^

In the present study, we demonstrated that high level of TANs was associated with poor clinical outcomes. TANs may contribute to suppressing cytotoxicity of CD8^+^ T cells under JAG2 dependent manner, which can be overcome by the Notch inhibitor, LY3039478. High JAG2^+^TANs can be served as a marker for the selective treatment of LY3039478 in patients with EOC.

## Supplementary information

Supplementary materials

## Data Availability

The datasets used and/or analysed during the current study are available from the corresponding author on reasonable request.
