# Supplementary material for: Tumour-associated neutrophils orchestrate intratumoural IL-8-driven immune evasion through Jagged2 activation in ovarian cancer
Source: Br J Cancer. 2020 Aug 11;123(9):1404–16. doi: 10.1038/s41416-020-1026-0 (PMC7591527; doi:10.1038/s41416-020-1026-0)
Supplement: Supplementary file 1 — Supplementary materials [file 41416_2020_1026_MOESM1_ESM.docx]

**Supplementary figure 1**

a

b

c

d

**Supplementary figure 1. Representative images of CD66b, CD8a, IL-8, and JAG2^+^CD66b were shown. a-c** Immunohistochemical staining for CD66b (a), CD8a(b), and IL-8(c) in TME showing infiltration of neutrophils, CD8^+^ T cells and immune cells expressing IL-8 in TME. **d** Neutrophils expressing JAG2 were marked by immunohistochemical double staining (red for CD66b, brown for JAG2).

**Supplementary figure 2**

**
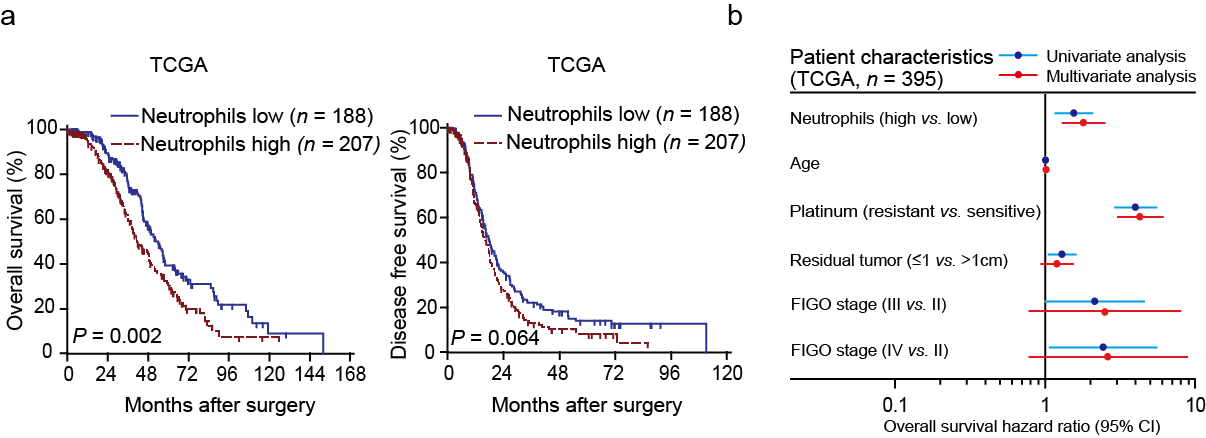
**

**Supplementary figure 2. Tumor-associated neutrophils correlate with poor clinical outcomes from TCGA.** **a** Kaplan-Meier plots for overall survival and disease-free survival of cases with ovarian cancer based on neutrophil proportion in the Cancer Genome Atlas cohort (TCGA). **b** Cox multivariate analysis identified the independent prognostic factors for overall survival in cases with ovarian cancer from TCGA.

**­Supplementary figure 3**


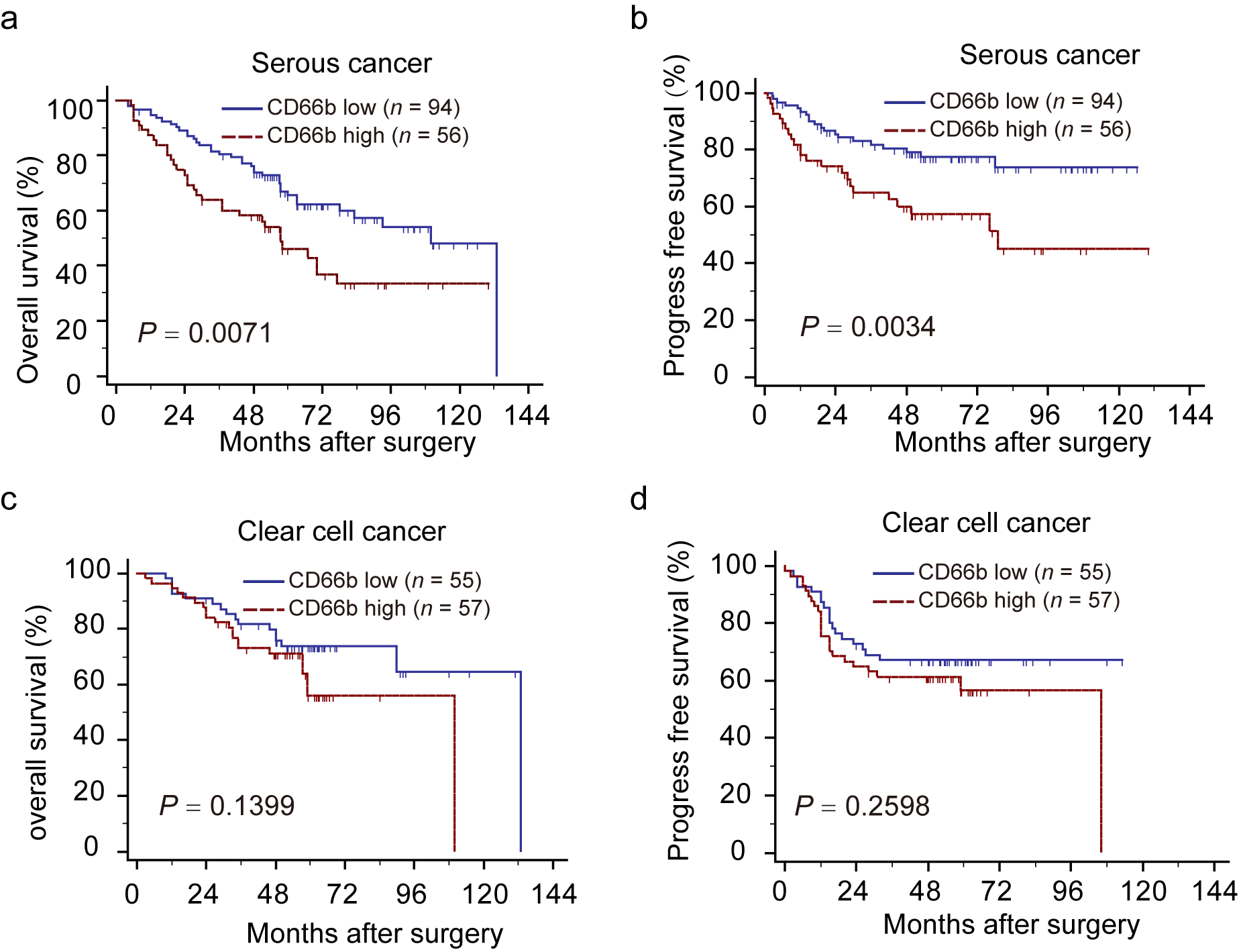


**Supplementary figure 3. K-M curves of CD66b cells in patients with different histology of EOC.** a. K-M curve of CD66b low and high group about OS in patients with serous ovarian cancer (*p*=0.0071). b. K-M curve of CD66b low and high group about PFS in patients with serous ovarian cancer (*p*=0.0034). c. K-M curve of CD66b low and high group about OS in patients with clear cell ovarian cancer (*p*=0.1399). d. K-M curve of CD66b low and high group about PFS in patients with clear cell ovarian cancer (*p*=0.2598).

**Supplementary figure 4**


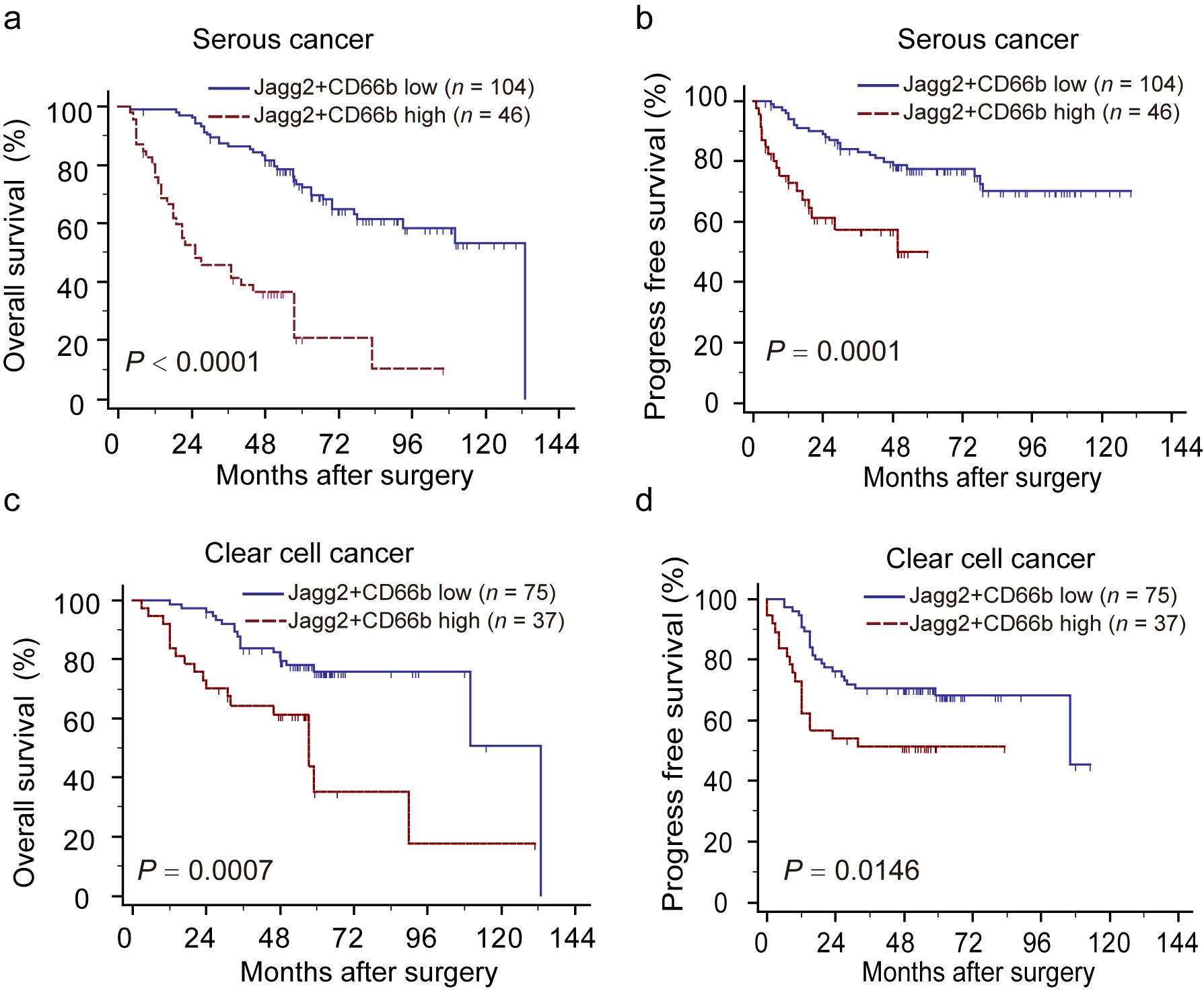


**Supplementary figure 4：K-M curves of JAG2^+^CD66b cells in patients with different histology of EOC.** a K-M curve of JAG2^+^CD66b low and high group about OS in patients with serous ovarian cancer (*p*<0.0001). a K-M curve of JAG2^+^CD66b low and high group about PFS in patients with serous ovarian cancer (*p*=0.00001). c. K-M curve of JAG2^+^CD66b low and high group about OS in patients with clear cell ovarian cancer (*p*=0.0007). d. K-M curve of JAG2^+^CD66b low and high group about PFS in patients with clear cell ovarian cancer (*p*=0.0146).

**Supplementary figure 5**

**
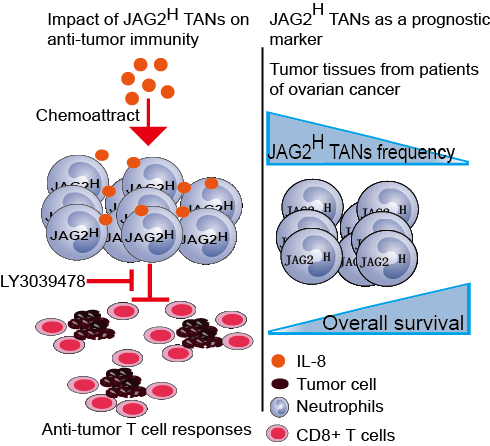
**

**Supplementary figure 5. Cartoon model of the relationship between IL8, CD8^+^ T cells, and TANs in EOC was shown.** Cartoon depicting that CXCL8 recruits JAG2^H^ TANs to the tumor microenvironment and confers immunosuppressive impact on anti-tumor T cell responses, which could be reversed by Notch inhibitor (LY3039478). In addition, JAG2^H^ TANs from tumor tissues may serve as a biomarker for patients of EOC with poor prognosis.

| **Supplementary table 1. Clinicopathological characteristics of training and validation cohorts.** | | | | | | | |
| --- | --- | --- | --- | --- | --- | --- | --- |
| **Group** | **Pooled(n=277)** | | **Training cohort（n=144）** | | **Validation cohort(n=133)** | | ***P*-value^a^** |
|  | ***No.*** | **%** | ***No.*** | **%** | ***No.*** | **%** |  |
| **Age (years)** | 57.0±10.7 | | 57.0±10.7 | | 56.7±10.7 | | 0.221 |
| **Ascites** |  |  |  |  |  |  | 0.260 |
| ≤20ml | 133 | 48.0 | 71 | 49.3 | 62 | 46.6 |  |
| >20ml | 123 | 44.4 | 57 | 39.6 | 66 | 49.6 |  |
| Unknown | 21 | 7.6 | 16 | 11.1 | 5 | 3.8 |  |
| **CA125** |  |  |  |  |  |  | 0.862 |
| ≤35mg/L | 30 | 10.8 | 16 | 11.1 | 14 | 10.5 |  |
| >35mg/L | 242 | 88.4 | 125 | 86.8 | 117 | 88.0 |  |
| Unknown | 5 | 1.8 | 3 | 2.1 | 2 | 1.5 |  |
| **Residual** |  |  |  |  |  |  | 0.497 |
| ≤10 mm | 227 | 81.9 | 121 | 84.0 | 106 | 79.7 |  |
| >10mm | 48 | 17.4 | 23 | 16.0 | 25 | 18.8 |  |
| Unknown | 2 | 0.7 | 0 | 0.0 | 2 | 1.5 |  |
| **Histology** |  |  |  |  |  |  | 0.250 |
| Serous cancer | 150 | 54.2 | 77 | 53.5 | 73 | 54.9 |  |
| Clear cell cancer | 112 | 40.4 | 55 | 38.2 | 57 | 42.9 |  |
| Others | 15 | 5.4 | 12 | 8.3 | 3 | 2.2 |  |
| **FIGO stage** |  |  |  |  |  |  | 0.150 |
| I | 65 | 23.5 | 37 | 25.7 | 28 | 21.1 |  |
| II | 32 | 11.6 | 22 | 15.3 | 10 | 7.5 |  |
| III | 148 | 53.4 | 71 | 49.3 | 77 | 57.9 |  |
| IV | 27 | 9.7 | 13 | 9.0 | 14 | 10.5 |  |
| Unknown | 5 | 1.8 | 1 | 0.7 | 4 | 3.0 |  |

^a^ *P*-value < 0.05 marked in bold font shows statistical significance.

**Supplementary table 2. clinicopathological characteristics of patients supplying fresh tumor tissue.**

| **NO.** | **Age** | **Histology** | **Grade** | **Stage** | **FCS** | **MACS** | **Ascites** |
| --- | --- | --- | --- | --- | --- | --- | --- |
| **1** | 38 | Clear cell cancer |  | IIC | + |  | + |
| **2** | 69 | Serous cancer | H | IIIC | + |  | + |
| **3** | 42 | Serous cancer |  |  | + |  | + |
| **4** | 48 | Serous cancer | L | IIIB | + |  | + |
| **5** | 63 | Serous cancer | H | IIA | + | + | + |
| **6** | 57 | Serous cancer | H | IIIC | + | + | + |
| **7** | 48 | Serous cancer | H | IIIC | + | + |  |
| **8** | 45 | Clear cell cancer |  | IIIC | + | + |  |
| **9** | 43 | Serous cancer | H | IVB | + |  |  |
| **10** | 50 | Serous cancer | H | IIIB | + |  |  |
| **11** | 79 | Clear cell cancer |  | IC | + |  |  |
| **12** | 52 | Serous cancer | H | IVB | + |  | + |
| **13** | 54 | Serous cancer | H | IIIC | + |  | + |
| **14** | 47 | Serous cancer | H | IIIC | + |  | + |
| **15** | 51 | Serous cancer | H | IIIC | + |  |  |
| **16** | 37 | Serous cancer | H | IIIC | + |  | + |
| **17** | 58 | Clear cell cancer |  | IC3 | + |  | + |
| **18** | 45 | Serous cancer | H | IIIC | + |  |  |
| **19** | 47 | Serous cancer | H | IB | + |  |  |
| **20** | 61 | Clear cell cancer |  | IIIC | + |  | + |
| **21** | 62 | Serous cancer | H | IIIC | + |  | + |
| **22** | 65 | Serous cancer | H | IC3 | + |  | + |
| **23** | 71 | Serous cancer | H | IIIC | + |  |  |
| **24** | 64 | Serous cancer | H |  | + |  |  |
| **25** | 64 | Serous cancer | H | IIIC | + |  | + |
| **26** | 63 | Serous cancer | H | IC3 | + |  | + |
| **27** | 53 | Serous cancer | H | IIIC | + |  |  |
| **28** | 48 | Endometrioid cancer | III | IC3 | + |  |  |
| **29** | 57 | Endometrioid cancer | H | IC3 | + |  |  |
| **30** | 49 | Clear cell cancer |  | IIIC | + |  |  |
| **31** | 51 | Serous cancer | H | IIIC | + |  |  |
| **32** | 66 | Clear cell cancer |  | IC3 | + |  |  |
| **33** | 62 | Serous cancer | H | IIIC | + |  | + |
| **34** | 51 | Serous cancer | H | IIIC | + |  | + |
| **35** | 51 | Serous cancer | H | IIIC | + |  |  |
| **36** | 54 | Serous cancer | H | IIA | + |  |  |
| **37** | 66 | Serous cancer | H | IVB | + |  |  |
| **38** | 66 | Serous cancer | H | IIIC | + |  | + |

**Supplementary table 3. Monoclonal antibodies used for flow cytometry**

| Material | Vendor | Catalog # |
| --- | --- | --- |
| Anti-human CD45-BV785 | Biolegend | 368528 |
| Anti-human CD8-FITC | Biolegend | 300912 |
| Anti-human CD8-APC | Biolegend | 301049 |
| Anti-human CD4-FITC | Biolegend | 317408 |
| Anti-human CD25-PE | Biolegend | 302606 |
| Anti-human Foxp3-Alexa Fluor647 | Biolegend | 320114 |
| Anti-human IFNγ-PE | Biolegend | 506507 |
| Anti-human Ki67-pacific blue | Biolegend | 350512 |
| Anti-human CD69-pacific blue | Biolegend | 310920 |
| Anti-human Granzyme B-PerCP/CY5.5 | Biolegend | 372212 |
| Anti-human CD66b-APC | Biolegend | 305118 |
| Anti-human DLL1-PE | Biolegend | 346404 |
| Anti-human DLL3-PE | R&D | FAB4315P |
| Anti-human DLL4-PE | Biolegend | 346506 |
| Anti-human JAG1-PE | BD bioscience | 565495 |
| Anti-human JAG2-PE | BD bioscience | 565548 |
| Anti-human PD1-FITC | Biolegend | 329904 |
| Anti-human TIM3-PE | Biolegend | 345006 |
| Anti-mouse CD45-BV785 | Biolegend | 103149 |
| Anti-mouse CD8-FITC | Biolegend | 100804 |
| Anti-mouse Ki67-PE | Biolegend | 151210 |
| Anti-mouse IFN-γ-PE | Biolegend | 505808 |
| Anti-mouse Granzyme B-PE | Biolegend | 372208 |
| Anti-mouse Ly6G-PE | Biolegend | 127608 |
| Anti-mouse CD11b-APC | Biolegend | 101212 |
| Live/Dead Fixable Violet Dead Cell Stain kit | ThermoFisher | L34966 |

**Supplementary table 4. Univariate and multivariate COX regression of clinicopathological characteristics and CD66b^+^ cells in training and validation cohorts.**

|  | |  | **OS** | | | | **PFS** | | | |
| --- | --- | --- | --- | --- | --- | --- | --- | --- | --- | --- |
|  |  |  | **univariate** | | **Multivariate** | | **Univariate** | | **Multivariate** | |
|  |  |  | ***P*** | **HR(95%CI)** | ***P*** | **HR(95%CI)** | ***P*** | **HR(95%CI)** | ***P*** | **HR(95%CI)** |
| **Pooled** | **Age** | | **0.031** | 1.024(1.002,1.045) | 0.190 | 1.015(0.993,1.037) | 0.495 | 0.992(0.970,1.015) |  |  |
|  | **Ascite** | | **0.040** | 1.472(1.017,2.131) | 0.174 | 1.353(0.875,2.095) | 0.468 | 0.855(0.561,1.304) |  |  |
|  | **Residual** | | 0.056 | 1.557(0.988,2.453) | 0.911 | 1.034(0.574,1.864) | **0.008** | 1.899(1.181,3.053) | **0.039** | 1.668(1.027,2.710) |
|  | **FIGO stage** | | **<0.001** | 3.608(2.173,5.991) | **0.001** | 2.597(1.476,4.570) | **0.000** | 2.682(1.594,4.511 | **0.006** | 2.140(1.249,3.664) |
|  | **CA125** | | 0.330 | 1.348(0.740,2.456) |  |  | 0.698 | 1.133(0.603,2.130) |  |  |
|  | **CD66b+** | | **0.001** | 1.881(1.291,2.739) | 0.066 | 1.533(0.973,2.379) | **<0.001** | 2.147(1.412,3.263) | **0.005** | 1.864(1.211,2.868) |
| **Training**  **cohort** | **Age** | | 0.238 | 1.018(0.988,1.049) | 0.509 | 1.010(0.980,1.042) | 0.601 | 0.992(0.962,1.023) |  |  |
|  | **Ascite** | | 0.459 | 1.236(0.705,2.166) |  |  | 0.483 | 0.802(0.433,1.485) |  |  |
|  | **Residual** | | 0.382 | 0.684(0.292,1.603) |  |  | 0.177 | 1.621(0.804,3.269) | 0.318 | 1.442(0.703,2.954) |
|  | **FIGO stage** | | **0.002** | 2.783(1.464,5.290) | 0.073 | 1.898(0.941,3.829) | **0.036** | 1.998(1.047,3.811) | 0.120 | 1.694(0.872,3.291) |
|  | **CA125** | | 0.714 | 1.188(1.464,5.290) |  |  | 0.822 | 0.906(0.384,2.138) |  |  |
|  | **CD66b+** | | **0.010** | 2.046(1.191,3.516) | **0.034** | 2.003(1.055,3.801) | **0.008** | 2.204(1.231,3.946) | **0.012** | 2.145(1.181,3.894) |
| **Validation cohort** | **Age** | | **0.046** | 1.031(1.000,1.063) | 0.135 | 1.024(0.993,1.056) | 0.694 | 0.994(0.962,1.026) |  |  |
|  | **Ascite** | | **0.028** | 1.885(1.069,3.324) | 0.444 | 1.298(0.666,2.532) | 0.689 | 0.882(0.477,1.631) |  |  |
|  | **Residual** | | **0.001** | 2.546(1.446,4.481) | 0.064 | 1.983(0.962,4.089) | **0.016** | 2.245(1.163,4.333) | 0.061 | 1.899(0.972,3.710) |
|  | **FIGO stage** | | **<0.001** | 4.786(2.037,11.248) | **0.008** | 4.333(1.461,12.847) | **0.002** | 4.288(1.676,10.973) | **0.019** | 3.233(1.215,8.598) |
|  | **CA125** | | 0.619 | 1.677(0.992,2.834) |  |  | 0.622 | 1.296(0.462,3.636) |  |  |
|  | **CD66b+** | | **0.053** | 1.667(0.992,2.834) | 0.730 | 1.117(0.596,2.092) | **0.022** | 2.025(1.108,3.703) | 0.216 | 1.485(0.794,2.799) |

**Supplementary table 5. Univariate and multivariate analyses of factors affecting OS and PFS in patients with ovarian cancer in TCGA cohort.**

| Variables | OS | | PFS | |
| --- | --- | --- | --- | --- |
|  | HR (95% CI) | *P* | HR (95% CI) | *P* |
| Univariate analyses | | | | |
| Age at diagnosis | 1.01(0.99-1.02) | 0.082 | 1.0(0.99-1.01) | 0.98 |
| FIGO stage  III vs II  IV vs II | 2.14(1.0-4.54)  2.43(1.08-5.49) | **0.049**  **0.034** | 1.95(1.14-3.34)  2.48(1.36-4.53) | **0.016**  **0.003** |
| Residual tumor(cm)  > 1 *vs* ≤ 1 | 1.09(0.79-1.51) | 0.597 | 1.15(0.87-1.51) | 0.328 |
| Platinum status  Resistant *vs* sensitive | 4.01(2.92-5.51) | **<0.001** | 22.98(15.21-34.73) | **<0.001** |
| Neutrophils  High *vs* low | 1.55(1.17-2.05) | **0.002** | 1.25(0.99-1.58) | 0.065 |
| Multivariate analyses | | | | |
| Age at diagnosis | 1.02(1.0-1.04) | **0.002** |  |  |
| FIGO stage  IV vs II |  |  | 1.48(1.02-2.14) | **0.038** |
| Platinum status  Resistant *vs* sensitive | 4.21(3.0-5.91) | **<0.001** | 24.29(15.73-37.49) | **<0.001** |
| Neutrophils  High *vs* low | 1.86(1.35-2.56) | **<0.001** |  |  |

**Supplementary table 6. Correlation of clinicopathological characteristics with JAG2^+^CD66b^+^ cells in training and validation cohorts.**

| **Characteristics** | **Pooled** | | | | **Training cohort** | | | **Validation cohort** | | |
| --- | --- | --- | --- | --- | --- | --- | --- | --- | --- | --- |
|  | **Low** | **High** | ***P*-value ^a^** | **Low** | | **High** | ***P*-value ^a^** | **Low** | **High** | ***P*-value ^a^** |
| **All patients** | 190 | 87 |  | 104 | | 40 |  | 86 | 47 |  |
| **Age (years)** | 57.0±10.7 | 57.0±10.6 | 0.509 | 57.0±10.8 | | 58.0±10.7 | 0.397 | 57.0±10.8 | 57.0±10.7 | 0.997 |
| **Ascites**  ≤20ml  >20ml  Unknown | 93  84  13 | 40  39  8 | 0.778 | 52  42  10 | | 19  15  6 | 0.955 | 41  42  3 | 21  24  2 | 0.768 |
| **CA125 (U/ml)**  ≤35  >35  NA | 24  162  4 | 6  80  1 | 0.147 | 12  89  3 | | 4  36  0 | 0.751 | 12  73  1 | 2  44  1 | 0.084 |
| **Residual** |  |  | 0.735 |  | |  | 0.481 |  |  | 0.301 |
| ≤10mm  >10mm  Unknown | 157  32  1 | 70  16  1 |  | 86  18  0 | | 35  5  0 |  | 71  14  1 | 35  11  1 |  |
| **Histology**  Serous cancer  Clear cell cancer  Others | 104  75  11 | 46  37  4 | 0.684 | 56  40  8 | | 21  15  4 | 0.904 | 48  35  3 | 25  22  0 | 0.379 |
| **FIGO stage** |  |  | **0.002** |  | |  | 0.083 |  |  | **0.037** |
| I  II  III  IV  NA | 55  24  94  13  4 | 10  8  54  14  1 |  | 32  16  48  7  1 | | 5  6  23  6  0 |  | 23  8  46  6  3 | 5  2  31  8  1 |  |

^a^ *P*-value < 0.05 marked in bold font shows statistical significance.

**Supplementary table 7. Univariate and multivariate COX regression of clinicopathological characteristics and JAG2^+^CD66b^+^ cells in training and validation cohorts**

|  |  | | **OS** | | | | **PFS** | | | |
| --- | --- | --- | --- | --- | --- | --- | --- | --- | --- | --- |
|  |  |  | **univariate** | | **Multivariate** | | **Univariate** | | **Multivariate** | |
|  |  |  | ***P*** | **HR(95%CI)** | ***P*** | **HR(95%CI)** | ***P*** | **HR(95%CI)** | ***P*** | **HR(95%CI)** |
| **Pooled** | | **Age** | **0.031** | 1.024(1.002,1.045) | 0.168 | 1.015(0.994,1.038) | 0.495 | 0.992(0.970,1.015) |  |  |
|  |  | **Ascite** | **0.040** | 1.472(1.017,2.131) | 0.158 | 1.360(0.887,2.084) | 0.468 | 0.855(0.561,1.304) |  |  |
|  |  | **Residual** | 0.056 | 1.557(0.988,2.453) | 0.560 | 1.192(0.660,2.153) | **0.008** | 1.899(1.181,3.053) | 0.017 | 1.820(1.115,2.973) |
|  |  | **FIGO stage** | **<0.001** | 3.608(2.173,5.991) | **0.015** | 2.047(1.148,3.650) | **0.000** | 2.682(1.594,4.511 | 0.014 | 1.980(1.147,3.416) |
|  |  | **CA125** | 0.330 | 1.348(0.740,2.456) |  |  | 0.698 | 1.133(0.603,2.130) |  |  |
|  |  | **JAG2^+^**  **CD66b^+^** | **<0.001** | 3.924(2.667,5.773) | **<0.001** | 3.533(2.246,5.558) | **<0.001** | 2.678(1.747,4.104) | 0.000 | 2.439(1.570,3.789) |
| **Training**  **cohort** | | **Age** | 0.238 | 1.018(0.988,1.049) | 0.670 | 1.007(0.976,1.038) | 0.601 | 0.992(0.962,1.023) |  |  |
|  |  | **Ascite** | 0.459 | 1.236(0.705,2.166) |  |  | 0.483 | 0.802(0.433,1.485) |  |  |
|  |  | **Residual** | 0.382 | 0.684(0.292,1.603) |  |  | 0.177 | 1.621(0.804,3.269) | 0.116 | 1.815(0.863,3.817) |
|  |  | **FIGO stage** | **0.002** | 2.783(1.464,5.290) | 0.179 | 1.637(0.797,3.360) | **0.036** | 1.998(1.047,3.811) | 0.183 | 1.579(0.806,3.094) |
|  |  | **CA125** | 0.714 | 1.188(1.464,5.290) |  |  | 0.822 | 0.906(0.384,2.138) |  |  |
|  |  | **JAG2^+^**  **CD66b^+^** | **<0.001** | 4.266(2.460,7.399) | **<0.001** | 3.947(2.064,7.550) | **0.004** | 2.437(1.336,4.445) | **0.004** | 2.566(1.358,4.846) |
| **Validation cohort** | | **Age** | **0.046** | 1.031(1.000,1.063) | 0.076 | 1.028(0.997,1.060) | 0.694 | 0.994(0.962,1.026) |  |  |
|  |  | **Ascite** | **0.028** | 1.885(1.069,3.324) | 0.249 | 1.463(0.766,2.793) | 0.689 | 0.882(0.477,1.631) |  |  |
|  |  | **Residual** | **0.001** | 2.546(1.446,4.481) | 0.083 | 1.872(0.992,3.804) | **0.016** | 2.245(1.163,4.333) | 0.083 | 1.811(0.925,3.544) |
|  |  | **FIGO stage** | **<0.001** | 4.786(2.037,11.248) | **0.043** | 3.155(1.038,9.588) | **0.002** | 4.288(1.676,10.973) | **0.031** | 2.959(1.105,7.922) |
|  |  | **CA125** | 0.619 | 1.677(0.992,2.834) |  |  | 0.622 | 1.296(0.462,3.636) |  |  |
|  |  | **JAG2^+^**  **CD66b^+^** | **<0.001** | 3.472(2.017,5.975) | **0.002** | 2.825(1.481,5.390) | **0.001** | 2.9281.586,5.405) | **0.018** | 2.142(1.139,4.028)) |
